# Efficacy of AI-delivered cognitive behavioral therapy interventions for anxiety and depressive symptoms: a systematic review

**DOI:** 10.1038/s41746-026-02744-w

**Published:** 2026-05-29

**Authors:** Wing Lam Tiffany Yip, Ya Yambao Yang, Zilu Lucia Wang, David Stuckler

**Affiliations:** 1 Nuffield Department of Primary Care Health Sciences, University of Oxford, Oxford, UK; 2 Department of Public Health, University of California, Davis, CA USA; 3 Department of Education, University of Oxford, Oxford, UK; 4 Department of Social and Political Sciences, University of Bocconi, Milan, Italy

**Keywords:** Outcomes research, Psychology, Software, Health care

## Abstract

Despite the availability of evidence-based treatments like CBT, many individuals still lack adequate care for anxiety and depression due to workforce shortages, systemic barriers, and cultural differences. AI-delivered CBT (AI CBT) offers a scalable solution to this issue, yet existing evidence remains fragmented and lacks diversity. This systematic review addresses this gap regarding AI CBTs. We searched Web of Science, PubMed, and PsycINFO for RCTs 2016 onward that evaluated AI CBT for anxiety or depressive symptoms. Of 208 studies screened, 16 met the inclusion criteria. Data was extracted and thematically synthesized. Study quality was assessed using the Cochrane Risk of Bias Tool 2. AI CBT showed limited efficacy for anxiety, with only 1 of 14 studies reporting significant improvements. Third-wave CBT and interventions targeting younger populations may show particular potential in reducing anxiety. For depressive symptoms, only 4 of 16 studies showed significant improvements. Across both conditions, highly preliminary evidence suggest that AI CBTs show less promise for older adults. Additionally, personalization and human support appeared to not be reliably associated with improved outcomes. Study quality also raised concerns. Overall, current AI CBTs require further refinement through high-quality and user-centered research. No funding went into this manuscript (PROSPERO: CRD42024615340).

## Introduction

Depression and anxiety are two of the leading contributors to the global burden of mental health disorders^1^. Depressive symptoms are characterized by ongoing profound sadness, loss of interest in daily activities, and chronic fatigue, whereas anxiety is defined by the anticipation of imagined threats, resulting in emotional distress and physiological tension^2^. Over the past few decades, the incidence of both conditions has escalated significantly, with the COVID-19 pandemic further exacerbating these challenges^1^.

Among a wide range of evidence-based treatments of depression and anxiety, Cognitive Behavioral Therapy (CBT) is the most empirically supported and considered to be the “gold standard of psychotherapy”^3,4^. Each historical stage, or “wave,” of CBT includes multiple therapies that emphasize different therapeutic components to produce change. Second-wave CBT therapies focus on changing maladaptive beliefs and cognitive distortions to reduce anxiety and depressive symptoms^4,5^. Third-wave CBT incorporates components of second-wave CBT but emphasizes mindfulness, acceptance, and values-based action^4^. These components help clients engage flexibly with distressing experiences to reduce anxiety and depression^4^. Yet, despite the availability of evidence-based treatments such as CBT, a large proportion of individuals do not receive adequate care^6^. Contributing factors include a shortage of trained professionals, financial and systemic barriers, and cultural differences in the acceptability of mental health interventions^7^.

To address these service limitations, Artificial Intelligence (AI)-enabled interventions have emerged as a potentially transformative approach to mental healthcare. Although AI lacks a universally accepted definition, it is generally described as a system capable of interpreting external data, learning from it, and applying this learning to achieve specific goals with adaptive flexibility^8^. AI’s capacity to process large-scale data, identify complex behavioral patterns, and generate personalized responses has made it an appealing tool for mental health interventions. Often, AI-delivered CBT is delivered in the form of a “chatbot”, which are computer programs designed to mimic human conversation^9^. In practice, users can interact with a chatbot like how they interact with another person, either through a text-based interface or a voice chat with an embodied avatar. In AI-delivered CBT, the level of personalization in user interactions depends critically on the technological sophistication of the AI system.

At the most basic level, rule-based systems rely on pre-scripted dialog trees and fixed algorithms, guiding users through structured CBT modules with little flexibility^10^. In practice, if a user tells the AI CBT chatbot that they are feeling anxious today, the chatbot will tap into a script library and send them a custom CBT-based anxiety exercise that is pre-written by professionals. This tailoring occurs at the population level: users in the group receive similar scripted content, rather than individualized responses that adapt dynamically to evolving needs^10^. As such, personalization remains limited.

To increase personalization, engineers use natural language processing (NLP) on top of rule-based AI to understand user input, allowing the system to tailor responses accordingly. Keyword-based NLP matches user inputs to a library of pre-written messages^11^. In practice, the AI chatbot identifies keywords in the users’ inputs and finds pre-written CBT-based messages that correspond to what they are saying. This allows for more variability in conversation, but interactions remain formulaic and constrained by pre-defined response banks^11^. Predictive machine learning–based NLP represents a further step toward personalization. These systems analyze language patterns to infer intent or emotion, enabling moderate personalization^11^. In practice, when the user tells the AI chatbot about their situation, the chatbot infers the user’s feelings and provides a relevant response from pre-written messages.

More recently, advanced AI-delivered CBT interventions rely on generative artificial intelligence (AI), particularly large language models (LLMs) like ChatGPT 4.0. Unlike earlier systems, these chatbots can generate context-aware responses in real time^10^. In practice, users communicate with these chatbots as if they were talking to a real human being. Mental health interventions delivered by these AI systems thus have the capability to deliver the most tailored and personalized interactions at present^10^.

While the potential of personalization for AI CBT interventions is frequently praised, research findings have been mixed. Some studies have reported favorable outcomes in feasibility, user satisfaction, and symptom reduction, whereas others have noted that users find AI CBT chatbots repetitive or lacking in emotional authenticity^12–15^. Past systematic reviews, while rigorous and high-quality, have evaluated AI CBT interventions primarily through the lens of earlier technologies, such as rule-based and simpler NLP systems^16,17^. This may overlook the growing potential of AI-based CBT interventions in light of the rapid evolution of machine learning and generative AI, particularly its implications for personalization, user engagement, and therapeutic efficacy. Furthermore, while early research tended to focus on Western populations and clinical depression or anxiety as primary outcomes, recent studies have expanded geographically across Asia, Europe, and the Americas, and broadened their scope to include other health conditions—such as substance use, insomnia, and chronic illness—with depression and anxiety as secondary outcomes^18–21^. These findings highlight the need for more updated research that captures these diverse and emerging outcomes.

Additionally, while AI CBT is frequently described as a promising solution by major healthcare providers, there is currently no high-quality systematic review examining rigorously designed studies to substantiate these claims of its efficacy. Engagement has been noted to increase with the integration of human support into AI interventions, but questions remain on whether this increased engagement translates to improved clinical efficacy^21^. Moreover, older studies cite novelty effects as drivers of mood improvement, further complicating interpretations of the success of AI CBT interventions^22^.

Given these developments, a new systematic review is warranted to assess the efficacy of AI-delivered CBT interventions in the current technological and clinical landscape. We must assess not only the outcomes of recent studies using advanced generative models, but also the design rigor, contextual implementation, and real-world feasibility of these interventions.

To address gaps in the current literature, our study conducted a systematic review with a thematic synthesis, offering a timely and rigorous examination of AI-delivered Cognitive Behavioral Therapy (AI CBT) interventions. Unlike previous reviews, which often focused on outdated forms of AI (like rule-based or predictive systems) or lacked methodological rigor, our review synthesized only high-quality, peer-reviewed randomized controlled trials (RCTs), ensuring a robust and credible evidence base. We captured the most recent and relevant literature, with most included studies published within the past few years, thereby reflecting the rapidly evolving landscape of AI applications in mental health care. Furthermore, we included studies across a diverse range of populations and clinical contexts to capture the full picture of its efficacy. We also noted down the technological aspects of the AI CBT interventions, and how it relates to personalization. Our review also offers guidance for the design, implementation, and optimization of future AI CBT interventions and studies.

## Results

### Descriptive statistics

**Fig. 1:**
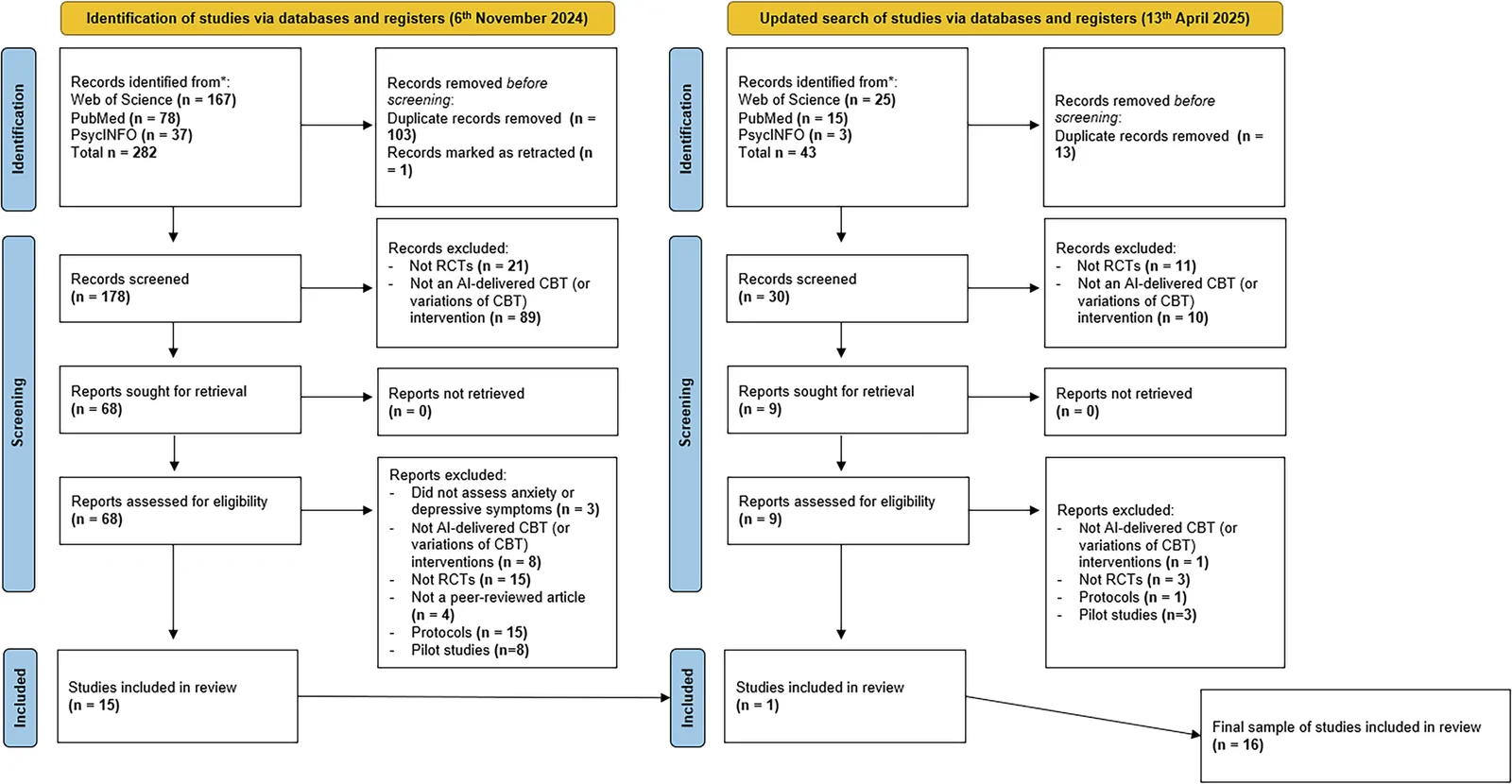
PRISMA Flow chart.

Our systematic review identified 16 articles published between 2018 and 2025 that examined Artificial Intelligence–delivered Cognitive Behavioral Therapy (AI CBT) interventions using a RCT design, targeting anxiety and/or depressive symptoms^18,21,23–36^. These studies covered 2875 participants across 10 regions, with most studies from the United States (*n* = 5). The included studies spanned 2 regions in Asia, 7 regions in Europe, and 1 region in North America. For anxiety symptom measures, the most used tool was the Generalized Anxiety Disorder-7 (GAD-7; *n* = 7). For depressive symptom measures, the most used tool was the Patient Health Questionnaire-9 (PHQ-9; *n* = 8). Most studies utilized AI interventions with second-wave CBT components (restructuring negative thoughts and behaviors), with only 3 studies using third-wave CBT components (acceptance-based and mindfulness methods). There were multiple modes of delivery for AI CBT interventions: Instant messenger app (*n* = 6), mobile app (*n* = 5), and online website platform (*n* = 5). The AI CBT interventions used a mixture of rule-based AI (*n* = 16), keyword-based NLP (*n* = 5), predictive machine learning-NLP (*n* = 4), machine-learning based voice-recognition (*n* = 1), and generative AI (*n* = 1). Control groups mostly consisted of a non-CBT AI (*n* = 4) or waitlist control conditions (*n* = 5), and others utilized bibliotherapy (*n* = 3), a self-guided intervention (*n* = 3), or no intervention (*n* = 3). The full extraction sheet and quality assessment can be found in Supplementary Data 1. Our PRISMA Flow chart is also shown in Fig. 1 below.

It is important to note that not all studies used an intention-to-treat (ITT) analysis when comparing the AI CBT intervention and control groups, which analyze all randomized participants^37^. Some studies only used a completer or per-protocol analysis, which only included individuals who completed the intervention and/or satisfied all pre-defined criteria when completing the intervention^37^. Some studies provided a completer or per-protocol analysis as Supplementary Information.

### Operationalizing personalization levels

For the purposes of this synthesis, we classified interventions as having higher or lower levels of personalization based on features reported in the extraction table (Supplementary Data 1): input type, NLP sophistication, and whether it incorporated data-driven feedback. For example, AI CBTs considered as having relatively higher personalization in this synthesis included those that used predictive or deep learning-based NLP, generative AI, and/or data-driven feedback with free-form input. AI CBTs considered as having relatively lower personalization were primarily rule-based AI with structured or keyword-matched input. As reporting of the AI CBT interventions were not standardized across studies, these distinctions are relative within the included studies rather than fixed thresholds.

### Impact of AI CBT interventions on anxiety

AI CBT interventions were mostly ineffective in reducing anxiety symptoms compared to control. Using an ITT analysis, only 1 out of 14 AI CBT interventions were effective in reducing anxiety symptoms compared to the control group. This single effective study featured an AI CBT intervention characterized by no human input, second-wave CBT principles, rule-based AI with predictive machine learning–based NLP, and a target population of university students with depression. In this study, a univariate ANCOVA revealed that the chatbot-enhanced self-help intervention showed a significant advantage to bibliotherapy control in terms of the reduction of anxiety with a low effect size (*d* = 0.30)^33^. When we include completer or per protocol analyses, 5 out of 14 AI CBT interventions were effective in reducing anxiety symptoms compared to the control group. These 5 AI CBT interventions used rule-based AI with predictive machine learning–based NLP (*n* = 2), rule-based AI only (*n* = 3), or Generative AI (*n* = 1; the same study used 2 versions of AI CBTs with two types of AI), second-wave CBT (*n* = 2) or third-wave CBT (*n* = 3), had no human input (*n* = 3) or human input (*n* = 2), and targeted diverse populations (university students with depression (*n* = 1), university students (*n* = 1), adolescents with depressive symptoms (*n* = 1), eighth-grade adolescents (*n* = 1), adults not in therapy (*n* = 1)).

This review suggests that AI CBT interventions with third-wave CBT components (Acceptance and Commitment Therapy, Mindfulness) may hold particular promise in supporting individuals with anxiety. For example, when we include completer or per protocol analyses, all AI CBT interventions utilizing third-wave CBT components were effective in reducing anxiety symptoms, which was 3 out of 5 studies using a per protocol analysis^24,31,32^. However, it is important to note that the current evidence is not sufficient to support firm conclusions. In the Tess intervention, third-wave CBT interventions were delivered as part of an eclectic approach that also incorporated CBT, mindfulness-based therapy, emotionally focused therapy, motivational interviewing, self-compassion therapy, and interpersonal psychotherapy^24^. The system adapted to users’ responses, providing more of the approaches that participants found helpful^24^. Consequently, some users may have received minimal exposure to third-wave components if they preferred other techniques, making it unclear how extensively these elements were delivered^24^. In addition, the two studies delivering the Youth Compass Plus intervention, which utilized third-wave CBT, drew from the same dataset. This means that the participant pool, study procedures, and data collection time points were largely overlapping. Therefore, while these studies begin to show promise in third-wave AI CBT interventions in reducing anxiety, the evidence remains preliminary. Further research is needed to explore whether AI interventions with third wave CBT are more effective than other AI CBT approaches.

Moreover, our synthesis raises a speculative point that AI CBT for anxiety may be more effective among younger compared to older populations. We observed that among the 5 studies demonstrating a significant reduction in anxiety, participants were predominantly younger, including 2 studies on university populations, 2 on adolescents, and 1 with a mean participant age of 31 years. On the other hand, the two AI CBT interventions designed exclusively for older populations (aged 50 or above) were not found to be effective in reducing anxiety symptoms. For example, the AI CBT intervention TEO (Therapy Empowerment Opportunity) was ineffective in reducing anxiety among older active workers in both Generalized Anxiety Disorder (GAD-7) and Symptom Checklist-90 (SCL-90) scales^27^. However, as no formal meta-analysis was conducted, these findings must be interpreted with caution. Moreover, the limited number of studies with older adult populations, along with the small sample sizes of those studies, makes it inappropriate to generalize these findings^25,27^. It is also important to note that the efficacy of AI CBT in younger populations was not uniform, as several studies with young adult samples also reported non-significant results^23,26,28^. Taken together, these findings suggest that there may be a preliminary signal worth investigating.

### Impact of AI CBT interventions on depressive symptoms

There were mixed results for AI CBT interventions in reducing depressive symptoms. Using ITT analyses, 4 out of 16 AI CBT interventions were effective in reducing depressive symptoms compared to the control group. The 4 AI CBT interventions all used rule-based AI (rule-based AI with predictive machine learning-based NLP (*n* = 2), rule-based AI only (*n* = 1)), second-wave CBT (*n* = 4), had no human input (*n* = 4), and targeted diverse populations (college students (*n* = 1), university students with depressive symptoms (*n* = 1), people with subclinical insomnia (*n* = 1), adults not in therapy (*n* = 1)). When we include completer or per protocol analyses, 7 out of 16 AI CBT interventions were effective in reducing depressive symptoms compared to the control group. These 7 AI CBT interventions used rule-based AI (rule-based AI with predictive machine learning-based NLP (*n* = 3), rule-based AI only (*n* = 3)), second-wave CBT (*n* = 5) or third-wave CBT (*n* = 2), had no human input (*n* = 6) or included human input (*n* = 1), and targeted diverse populations (college students (*n* = 2), university students with depressive symptoms (*n* = 1), people with subclinical insomnia (*n* = 1), adults not in therapy (*n* = 1), IBS patients (*n* = 1), eighth-grade adolescents (*n* = 1)).

To start with, we observed that AI CBT interventions may not be effective in reducing depressive symptoms among older populations compared to control. 2 out of 16 AI CBT interventions were designed for older populations (50 or above), and all of them were ineffective in reducing depressive symptoms. For example, a study examining the effects of the AI-CBT intervention MYLO on older adults with emotional distress found no significant differences in depressive symptoms between the intervention and control groups^25^. However, these findings should be interpreted with caution, given the absence of a formal meta-analyses and the limited number of studies and small sample sizes involving older adults. This observation points to potential age-related differences that merits further investigation.

### Technological and supplementary factors

Furthermore, we observed that AI CBT interventions with higher levels of personalization, powered by more technologically advanced models, may not be more effective in reducing anxiety nor depressive symptoms. Both effective and ineffective groups of studies had a mixture of more personalized (free-form text input/predictive machine-learning NLP/Generative AI/data-driven feedback)^23,24,27,29,33,35^, and less personalized (structured input/keyword-based NLP/Rule-based AI)^18,21,25,26,28,30–32,34–36^, AI CBT interventions.

We also observed that AI CBT interventions with added human input may not be more effective in reducing both anxiety and depressive symptoms. 5 out of 16 studies had added human input in their AI CBT intervention, including support from trained students, research assistants, health coaches, therapists, or integration with traditional therapy. However, there were no clear differences in outcomes between AI CBTs with and without added human support. For example, in an AI CBT intervention for insomnia, there was no significant difference in depressive nor anxiety symptoms between all guided dCBTi (AI groups), which included groups with human support, and dCBTi-unguided (control group)^21^.

Nonetheless, due to the absence of a meta-analysis and the small number of studies included, definitive conclusions remain premature.

### Risk of bias assessment

We used the Cochrane Risk of Bias Tool 2 in our quality assessment of the papers. The full quality assessment is shown in Supplementary Data 1. All studies were rated as having some concerns or high risk of bias, largely due to very high attrition rates. Additionally, several studies had methodological limitations, such as the absence of ITT analyses, the lack of pre-registered protocols or analysis plans, and lack of full blinding.

## Discussion

Our systematic review highlights a series of important findings. Overall, while there were signs of potential for both anxiety and depression alleviation, the results were mostly null. For anxiety, only 1 out of 14 AI-delivered CBT interventions showed efficacy when analyzed using ITT. For depressive symptoms, only 4 out of 16 AI CBT interventions reported significant improvements when analyzed using ITT. Of note, based on the evidence available, neither greater personalization nor added human input appeared to enhance AI CBT interventions for anxiety or depression. Similarly, highly preliminary evidence suggests that AI CBT interventions may be ineffective on older populations, although conclusions are provisional and require further investigation. Our quality assessment revealed that all papers had “some concerns” or “high risk of bias” due to the high attrition rate of participants. This suggests that the conclusions from this study are tentative and should be interpreted cautiously until more robust evidence becomes available.

One potential finding of this review is that AI-delivered third-wave CBT is more effective than second-wave CBT in reducing anxiety symptoms. However, recent meta-analyses found that second- and third-wave CBT approaches are equally effective for anxiety^38,39^. This suggests that the difference we observed could be due to how well each approach fits with AI delivery, rather than the therapy itself. Both second- and third-wave CBT involve personalized dialog, Socratic questioning, and can be delivered as transdiagnostic approaches. However, they differ significantly in the specific mechanisms used to facilitate change. The core therapeutic process of second-wave CBT relies on cognitive restructuring, which is analyzing, challenging, and reframing irrational thoughts. This requires the AI to possess a high level of reasoning to successfully deconstruct the logic of a client’s deep-seated cognitions^4,40^. However, even the most advanced AI models lack genuine logical deduction, relying instead on pattern recognition from their training data^41^. Their hallucinations, logically false conclusions, and unreliable outputs make effective second-wave delivery difficult. In contrast, third-wave strategies, such as cognitive defusion found in ACT, focuses on altering the client’s relationship to their thoughts rather than engaging in a complex logical analysis of the thought’s content^40^. Additionally, third-wave approaches also use other techniques to achieve therapeutic change, like emotional acceptance, detachment from unhelpful thoughts, and grounding in the present moment, most of which do not require complex logical reasoning needed in restructuring^4^. These third-wave exercises are often highly procedural and structured, leveraging the AI’s strength in generating step-by-step guidance^41^. Taken together, these observations raise a speculative hypothesis: third-wave CBT strategies may be comparatively more feasible for AI to deliver. Future research should empirically test this hypothesis by directly comparing how second- and third-wave CBT components perform with AI delivery.

Our preliminary findings begin to suggest that greater personalization from more advanced AIs may not alter the efficacy of AI CBT interventions, even though earlier work has linked it to higher engagement^42–44^. This may be due to the way advanced technology was implemented. For instance, some features may have felt surface-level or not truly responsive to users’ emotional needs^45^. Specifically, in our included studies that also examined acceptability, users described the AI CBT interventions as inflexible, tedious, and mechanical^29,35^. Issues included glitches, lagging, incoherent responses, and the failure to recall earlier conversation points^29,35^. Participants wanted smoother dialog, more emotional responsiveness, and better system performance^29,35^. Furthermore, it is also possible that the type of AI used was not well-suited to delivering the psychological intervention effectively. While personalization may enhance initial engagement, it does not necessarily lead to better clinical outcomes if the therapeutic content itself is not aligned with users’ needs. Future research could look into why current personalization strategies may not consistently improve anxiety and depressive symptoms and explore which types of AI work best with different therapy approaches.

While human input may help boost engagement by encouraging participants to complete the intervention^44^, this does not necessarily translate into improved efficacy. In some cases, users may view human support more as a form of accountability than as something that enhances the therapeutic experience. While all human support in the reviewed studies was therapeutic in nature (e.g., delivered by therapists, health coaches, and trained research assistants or psychology students), intensity varied widely. However, given the small number of supported studies (*n* = 5) and absence of a meta-analysis, identifying a specific threshold was not feasible without risking false-positive conclusions. Consequently, drawing definitive conclusions regarding the efficacy of added human support is premature. Future studies should systematically compare different intensities and levels of therapeutic human involvement to clarify whether such a threshold exists and how AI can work with human support to enhance therapeutic outcomes.

Preliminary evidence from our review points to potentially limited efficacy of AI CBT interventions in older adults. This may be linked to factors such as lower digital literacy, reduced familiarity or comfort with AI technologies, or a lack of trust in automated mental health tools. One study suggested that voice-based assistants may offer a more intuitive and accessible modality for older adults, potentially improving engagement and outcomes in this demographic^46^.

As with all systematic reviews, our study is subject to several important limitations. First, it was not possible to conduct a meta-analysis for this study. This is because most studies had at least some risk of bias, owing to extremely high attrition rates and poor statistical design. Combining low-quality studies in meta-analysis could create an artificial sense of certainty. Further, although all interventions were deploying AI and CBT, their content and delivery varied markedly, which a meta-analytic approach would conflate as though it were one standardized intervention. Second, AI for mental health is rapidly evolving, drawing upon new technologies and advances in AI. While we were unable to evaluate heterogeneity based on AI-model type (e.g., ChatGPT vs. DeepSeek or BERT vs. RASA), it is possible that performance may continue to improve with technological advances.

Limitations also emerged from existing studies. One is that many studies were of low methodological quality. All studies included had some concerns or high risk of bias due to attrition rates, poor statistical design, and lack of pre-registration among others. Additionally, some studies only used a per protocol analysis, which would lose the benefits of randomization and lead to an overestimation of intervention efficacy. Second, most of the AI CBT interventions had high attrition rates and were not more effective compared to control conditions. When interpreting the results of this review, it is important to note that we discussed findings in terms of both ITT and the often more “favorable” per-protocol analyses. ITT and per protocol analyses have significant discrepancies, as high attrition carries a high risk of bias and perceived efficacy may be inflated in non-ITT reporting. High attrition rates also raise questions about the engagement and usability of these interventions in real-world settings. Third, not all studies provided sufficiently detailed information on the AI CBT intervention, such as its technological design, sample dialogs, user interface and features to enable direct comparisons (see point about meta-analysis above). Greater transparency and standardization are needed in reporting this new generation of AI interventions. Fourth, despite our aim to include more generative AI studies, only one RCT involving GenAI was identified in our review. Despite the widespread availability of GenAI for several years, our search only retrieved pilot intervention studies involving this technology. This may reflect challenges inherent to GenAI, as its unreliable outputs (“hallucinations”) can create safety concerns and slow the development of GenAI-driven CBT interventions^47^. Nevertheless, we were able to include AI systems using newer technologies, which extends the scope of prior reviews. Finally, we must acknowledge publication bias, which means that positive findings are more likely to be published than negative ones. As a result, our systematic review may overestimate AI CBT’s efficacy, especially since novelty and enthusiasm in the AI field further drive selective publication.

Despite these limitations, our study has some key strengths. First, we included a diverse range of studies from various countries examining different populations, which was a key limitation in previous reviews. Notably, our review included a reasonably balanced number of articles from Asia, Europe, and North America, although no studies were identified from South America, Africa, or Oceania. The AI CBT interventions also varied widely in terms of the type of AI used, the level of personalization, the mode of delivery (e.g., website, mobile app), and the specific form of CBT applied. This diversity highlights the comprehensiveness of our findings. Second, this review only included RCTs and excluded pilot studies and those that were not peer-reviewed, to support stronger causal inference and enhance the potential for more reliable and valid conclusions^48^.

Despite the growing enthusiasm around AI-delivered CBT and the widespread push from healthcare providers for its adoption, this review highlights that these interventions still require significant refinement. The promise of revolutionizing mental health care remains largely unmet at this stage. Simply increasing personalization through advanced large language models or supplementing AI with human support does not automatically lead to better outcomes. More research is urgently needed to understand the reasons behind high attrition rates, determine which forms of CBT are best suited for AI delivery, and explore how these technologies can be optimized to genuinely enhance mental health care. Additionally, modifications could be made to enhance therapeutic outcomes in AI CBT interventions. For example, one study suggested that voice assistant-based AI CBT was more user-friendly for older adults than chatbot or text-style AI CBT^46^. Additionally, having gamification elements may increase efficacy and engagement, and including social support features may be more attractive to women^49^.

Our systematic review highlights several promising avenues for future research. First, there is a need to explore a broader range of CBT modalities delivered via different types of AI to understand which approaches are most effective in different contexts. Second, future studies should prioritize methodological rigor, including standardizing reporting of AI CBT interventions, improving research design and adopting strategies to reduce attrition rates. Third, future studies evaluating the efficacy of the newest AI-based CBT interventions for anxiety and depression are needed, with particular attention to user-centered design principles to improve real-world adoption. Finally, we also need to further investigate how AI CBT performs across different age groups. Future studies should examine which populations benefit most, whether adaptations are needed for specific age cohorts, and whether the efficacy of AI CBT interventions is truly consistent across diverse demographic groups.

This study also highlights several key implications for mental health practice and policy. Considering the findings of this study, it is important to develop a further understanding of the application of AI CBT interventions in real-world settings before and during widespread adoption in clinical contexts. In policymaking, it is also crucial to consider whether AI CBT, despite its promises for personalization, could be adopted for all populations. For instance, while definitive conclusions cannot be drawn, AI CBT may be less effective in older populations. Policymakers should prioritize rigorous, user-centered testing of AI CBT interventions to generate evidence that guides their development and integration. This approach can help prevent them from falling into the trap of techno-determinism and ensure that these tools are genuinely beneficial across diverse contexts.

Even though this review focused on the efficacy of AI-delivered CBT for anxiety and depression, it is also important to acknowledge the potential risks associated with its use. GenAI models are often fine-tuned with human feedback to optimize user satisfaction, which can sometimes lead to uncritical validation of harmful behaviors. Since AI CBT is delivered to vulnerable populations, including those at risk of suicide or eating disorders such as bulimia, there is a need to ensure user safety. Therefore, future research must prioritize its responsible implementation in high-risk groups as well.

## Methods

### Design and protocol registration

We performed our search following the PRISMA (Preferred Reporting Items for Systematic Reviews and Meta-Analyses) guidelines^50^. Our PRISMA Checklist can be found in Supplementary Table 1. We used the protocol published in the PROSPERO repository (CRD42024615340).

### Search strategy

We searched Web of Science, PubMed, and PsycINFO (via OVID) on 6^th^ November 2024. To ensure the inclusion of newly published literature, the search was updated on 13^th^ April 2025 using the same strategy. Our search strategy built upon previous systematic reviews^17,51^, operationalizing different permutations of each keyword: “AI”, “CBT”, “anxiety” and “depression”.

Furthermore, we aimed to capture the latest technologies used in AI-delivered CBT. The transformer architecture, invented in 2016, is an AI model designed to understand context and meaning more effectively^52^. In AI-delivered CBT, transformers underpin more advanced AI systems, including predictive machine-learning based NLPs and LLMs, although many existing CBT applications still rely on earlier AI technologies. Hence, the scope of this search was limited to publications from 2016 onward. The search strategies can be found in Supplementary Table 2.

Our initial search (6th November 2024) identified a total of 282 articles: 167 articles in Web of Science, 78 articles in PubMed, and 37 articles in PsycINFO. Our updated search (13th April 2025) identified a total of 43 additional articles: 25 articles in Web of Science, 15 articles in PubMed, and 3 articles in PsycINFO. These articles were then imported into Zotero. 116 duplicate records and 1 retracted record were identified and removed, which left a total of 208 articles for screening and eligibility stages.

### Inclusion/exclusion criteria

We established a series of inclusion and exclusion criteria using the PICOTS framework, as follows: (1) Population: all demographic groups were considered eligible; (2) Intervention: only studies utilizing AI-delivered CBT interventions, or variations of CBT, were included. In this study, AI-delivered CBT interventions were defined as software-based agents or bots using AI models to administer and/or conduct the therapy, or components of the therapy, in order to achieve therapeutic outcomes for participants; (3) Comparator: studies with any form of comparison were included, whether that was standard care or an alternative CBT intervention; (4) Outcome: any outcomes related to anxiety or depressive symptoms qualified, whether measured by self-reported surveys, objective indicators (e.g., passive sensing systems like audio or visual data), or third-party assessments; (5) Time: Only studies published on or after 2016-01-01 till now were included; (6) Study Design: only RCTs were included. Only peer-reviewed articles were included. This review also excluded pilot studies to ensure high quality of data. A detailed description of the eligibility criteria based on PICO, along with examples, is provided in Supplementary Table 3.

We then screened the remaining citations and abstracts in two stages: first by title and abstract, then through a full-text review. Out of the 208 articles screened, we excluded 32 studies because they were not RCTs and 99 because they did not investigate the intervention (AI CBT) relevant to our research question. All remaining records were retrieved for a full-text review, resulting in 77 articles for the full-text review stage. Upon reviewing the full texts, we excluded 3 articles for not assessing anxiety or depressive symptoms, 9 articles for not involving an AI-delivered CBT (or variations of CBT) intervention, 18 articles for not being a RCT, 4 articles for not being peer-reviewed, 16 study protocols, and 11 pilot studies. This left us with 16 articles for the final review.

### Reviewer consensus

Two reviewers (W.L.T.Y., Z.L.W.) assessed all articles for inclusion in the study. Each reviewer independently determined whether the articles were relevant, and their decisions were compared to check for consistency. If the two reviewers disagreed on whether an article should be included, a third reviewer (Y.Y.Y.) was consulted to make the final decision.

For both abstract and full-text review stages, we used Cohen’s kappa to assess interrater reliability^53^. In the abstract screening stage, there was “almost perfect agreement” between the two reviewers (*κ* = 0.865, 95% CI: 0.788–0.942). In the full-text review stage, there was “perfect agreement” between the two reviewers (*κ* = 1.000, 95% CI: 1.000–1.000).

### Data extraction

We created a data extraction form and trialed it on a few studies to ensure that it is reliable and reproducible. These data points were then extracted from the eligible studies and shown in Supplementary Data 1: publication details (author, title), AI CBT intervention details (country, intervention length, type of RCT, RCT population, AI CBT tool(s), mode of AI-delivery, type of Artificial Intelligence (AI), type of CBT intervention, human input, AI CBT group size (allocated)), control group details (control group, control group size (allocated)), and outcome measures and results for anxiety and depression. Reporting of the “Type of Artificial Intelligence (AI)” varied across studies, but all provided enough technical and functional detail for categorization based on the types outlined in the introduction. Furthermore, when studies did not conduct analyses using an ITT approach, this was explicitly stated and results were reported as per-protocol analyses.

We used the Cochrane Risk of Bias 2 tool to evaluate potential bias across included RCTs^54^. This tool assesses five key domains of bias related to the randomization process, deviations from intended interventions, missing outcome data, outcome measurement, and selection of reported results. Each study was rated as having low risk, some concerns, or high risk of bias. All assessments followed Cochrane’s recommended procedures.

We organized and tabulated the extracted data of individual studies in Excel. We thematically synthesized the data to identify common patterns and differences, as a meta-analysis was not feasible.

## Supplementary information

Supplementary Information

Supplementary Data 1

## Data Availability

The dataset supporting this review, including the extraction table and quality assessment, is provided in the Supplementary Information. The list of screened but excluded studies can be shared upon request from the corresponding author.
