# Supplementary material for: Efficacy of AI-delivered cognitive behavioral therapy interventions for anxiety and depressive symptoms: a systematic review
Source: NPJ Digit Med. 2026 May 29;9:750. doi: 10.1038/s41746-026-02744-w (PMC13631303; doi:10.1038/s41746-026-02744-w)
Supplement: Supplementary file 1 [file 41746_2026_2744_MOESM1_ESM.pdf]

Supplementary Information

Effectiveness of AI-delivered Cognitive Behavioral Therapy Interventions for Anxiety and Depressive Symptoms: A Systematic Review

Yip, Wing Lam Tiffany[1*], Yang, Ya[2], Wang, Zilu (Lucia)[3*] & Stuckler, David[4]

[1] – University of Oxford, Nuffield Department of Primary Care Health Sciences

[2] – University of California, Davis, Department of Public Health

[3] – University of Oxford, Department of Education

[4] – University of Bocconi, Department of Social and Political Sciences

* - corresponding author

Radcliffe Primary Care Building, Radcliffe Observatory Quarter,

Oxford OX2 6GG

United Kingdom

E) reub0434@ox.ac.uk (winglamtiffany@gmail.com)

T) +44 7747079842

Table of contents

Supplementary Table 1: PRISMA Checklist 3

Supplementary Table 2: Search strategy 6

Supplementary Table 3: Eligibility criteria for study inclusion 11

Supplementary Data 1: Extraction table and Quality assessment 12

Supplementary Table 1: PRISMA Checklist

Section and Topic	Item #	Checklist item	Location where item is reported
**TITLE**			
Title	1	Identify the report as a systematic review.	P.1
**ABSTRACT**			
Abstract	2	See the PRISMA 2020 for Abstracts checklist.	P.2
**INTRODUCTION**			
Rationale	3	Describe the rationale for the review in the context of existing knowledge.	P.3-6
Objectives	4	Provide an explicit statement of the objective(s) or question(s) the review addresses.	P.6
**METHODS**			
Eligibility criteria	5	Specify the inclusion and exclusion criteria for the review and how studies were grouped for the syntheses.	P.19
Information sources	6	Specify all databases, registers, websites, organisations, reference lists and other sources searched or consulted to identify studies. Specify the date when each source was last searched or consulted.	P.19
Search strategy	7	Present the full search strategies for all databases, registers and websites, including any filters and limits used.	P.19; Supplementary Table 2
Selection process	8	Specify the methods used to decide whether a study met the inclusion criteria of the review, including how many reviewers screened each record and each report retrieved, whether they worked independently, and if applicable, details of automation tools used in the process.	P.20
Data collection process	9	Specify the methods used to collect data from reports, including how many reviewers collected data from each report, whether they worked independently, any processes for obtaining or confirming data from study investigators, and if applicable, details of automation tools used in the process.	P.21
Data items	10a	List and define all outcomes for which data were sought. Specify whether all results that were compatible with each outcome domain in each study were sought (e.g. for all measures, time points, analyses), and if not, the methods used to decide which results to collect.	P.21-22
	10b	List and define all other variables for which data were sought (e.g. participant and intervention characteristics, funding sources). Describe any assumptions made about any missing or unclear information.	P.21-22

Study risk of bias assessment	11	Specify the methods used to assess risk of bias in the included studies, including details of the tool(s) used, how many reviewers assessed each study and whether they worked independently, and if applicable, details of automation tools used in the process.	P.22
Effect measures	12	Specify for each outcome the effect measure(s) (e.g. risk ratio, mean difference) used in the synthesis or presentation of results.	P.19; Supplementary Table 4
Synthesis methods	13a	Describe the processes used to decide which studies were eligible for each synthesis (e.g. tabulating the study intervention characteristics and comparing against the planned groups for each synthesis (item #5)).	P.20
	13b	Describe any methods required to prepare the data for presentation or synthesis, such as handling of missing summary statistics, or data conversions.	P.22
	13c	Describe any methods used to tabulate or visually display results of individual studies and syntheses.	P.22
	13d	Describe any methods used to synthesize results and provide a rationale for the choice(s). If meta-analysis was performed, describe the model(s), method(s) to identify the presence and extent of statistical heterogeneity, and software package(s) used.	P.22
	13e	Describe any methods used to explore possible causes of heterogeneity among study results (e.g. subgroup analysis, meta-regression).	NA
	13f	Describe any sensitivity analyses conducted to assess robustness of the synthesized results.	NA
Reporting bias assessment	14	Describe any methods used to assess risk of bias due to missing results in a synthesis (arising from reporting biases).	P.21
Certainty assessment	15	Describe any methods used to assess certainty (or confidence) in the body of evidence for an outcome.	P.21
**RESULTS**			
Study selection	16a	Describe the results of the search and selection process, from the number of records identified in the search to the number of studies included in the review, ideally using a flow diagram.	P.7-8
	16b	Cite studies that might appear to meet the inclusion criteria, but which were excluded, and explain why they were excluded.	NA
Study characteristics	17	Cite each included study and present its characteristics.	Supplementary Table 4
Risk of bias in studies	18	Present assessments of risk of bias for each included study.	Supplementary Table 4

Results of individual studies	19	For all outcomes, present, for each study: (a) summary statistics for each group (where appropriate) and (b) an effect estimate and its precision (e.g. confidence/credible interval), ideally using structured tables or plots.	Supplementary Table 4
Results of syntheses	20a	For each synthesis, briefly summarise the characteristics and risk of bias among contributing studies.	P.8-12
	20b	Present results of all statistical syntheses conducted. If meta-analysis was done, present for each the summary estimate and its precision (e.g. confidence/credible interval) and measures of statistical heterogeneity. If comparing groups, describe the direction of the effect.	NA
	20c	Present results of all investigations of possible causes of heterogeneity among study results.	NA
	20d	Present results of all sensitivity analyses conducted to assess the robustness of the synthesized results.	NA
Reporting biases	21	Present assessments of risk of bias due to missing results (arising from reporting biases) for each synthesis assessed.	Supplementary Table 4
Certainty of evidence	22	Present assessments of certainty (or confidence) in the body of evidence for each outcome assessed.	Supplementary Table 4
**DISCUSSION**			
Discussion	23a	Provide a general interpretation of the results in the context of other evidence.	P.13-15
	23b	Discuss any limitations of the evidence included in the review.	P.15-16
	23c	Discuss any limitations of the review processes used.	P.15
	23d	Discuss implications of the results for practice, policy, and future research.	P.17-18
**OTHER INFORMATION**			
Registration and protocol	24a	Provide registration information for the review, including register name and registration number, or state that the review was not registered.	P.19
	24b	Indicate where the review protocol can be accessed, or state that a protocol was not prepared.	P.19
	24c	Describe and explain any amendments to information provided at registration or in the protocol.	P.19
Support	25	Describe sources of financial or non-financial support for the review, and the role of the funders or sponsors in the review.	P.23
Competing interests	26	Declare any competing interests of review authors.	P.23
Availability of data, code and other materials	27	Report which of the following are publicly available and where they can be found: template data collection forms; data extracted from included studies; data used for all analyses; analytic code; any other materials used in the review.	P.23

Supplementary Table 2: Search strategy

First search

Search conducted on 6th November 2024.

Source: Web of Science Core Collection

	Search strategy (Limit: 2016-01-01 to 3000-12-31)	Results
#1	TS=(anxiety OR "panic attack" OR anxious OR depress* OR "mood disorder*" OR "major depressive disorder" OR dysthymia OR "affective disorder*")	554,947
#2	TS=(NLP OR "Natural Language Processing" OR "large language model" OR LLM OR GPT OR chatbot OR "chat-bot" OR "machine learning" OR "artificial intelligence" OR AI OR "multimodal large language models" OR MLLM OR "conversational agent*" OR "conversational bot*" OR "conversational system*" OR "chatterbot*" OR "chatter bot*" OR "smartbot*" OR "smart bot*" OR "smart-bot*" OR "virtual coach*" OR "virtual agent*")	625,920
#3	TS=("Cognit* Behavio* Therap*" OR "Cognit* Therap*" OR "Behavio* Therap*" OR CBT OR "Dialect* Behavio* Therap*" OR DBT OR "Metacogniti* Therap*" OR "Mind Training" OR "Behavio* Activation" OR "Cognitive Restructur*" OR Cognit* Training* OR "Behavi* Training" OR "iCBT" or "I-CBT" OR "eCBT" OR "e-CBT" OR "cCBT" OR "c-CBT" OR "Acceptance and Commitment Therapy" OR "Acceptance Commitment Therapy" OR ACT)	691,077
#4	TS=(randomised OR randomized OR randomisation OR randomization OR "controlled clinical trial*" OR placebo* OR (random* AND (allocat* OR assign*)) OR (blind* AND (single OR double OR treble OR triple)))	799,902
#5	#1 AND #2 AND #3 AND #4	167

Source: PubMed

	Search strategy (AND 2016/01/01:3000/12/31[Date - Create])	Results
#1	("anxiety"[Title/Abstract] OR "panic attack"[Title/Abstract] OR "anxious"[Title/Abstract] OR "depress*"[Title/Abstract] OR "mood disorder*"[Title/Abstract] OR "major depressive disorder"[Title/Abstract] OR "dysthymia"[Title/Abstract] OR "affective disorder*"[Title/Abstract] OR "Depression"[Mesh] OR "Anxiety"[Mesh])	816,727
#2	("NLP"[Title/Abstract] OR "Natural Language Processing"[Title/Abstract] OR "large language model"[Title/Abstract] OR "LLM"[Title/Abstract] OR "GPT"[Title/Abstract] OR "chatbot"[Title/Abstract] OR "chat-bot"[Title/Abstract] OR "machine learning"[Title/Abstract] OR "artificial intelligence"[Title/Abstract] OR "AI"[Title/Abstract] OR "multimodal large language models"[Title/Abstract] OR "MLLM"[Title/Abstract] OR "conversational agent*"[Title/Abstract] OR "conversational bot*"[Title/Abstract] OR "conversational system*"[Title/Abstract] OR "chatterbot*"[Title/Abstract] OR "smartbot*"[Title/Abstract] OR "smart bot*"[Title/Abstract] OR "smart bot*"[Title/Abstract] OR "virtual coach*"[Title/Abstract] OR "virtual agent*"[Title/Abstract])	191,582

#3	"cognit* behavio* therap*"[Title/Abstract] OR "cognit* therap*"[Title/Abstract] OR "behavio* therap*"[Title/Abstract] OR "CBT"[Title/Abstract] OR "dialect* behavio* therap*"[Title/Abstract] OR "DBT"[Title/Abstract] OR "metacogniti* therap*"[Title/Abstract] OR "Mind Training"[Title/Abstract] OR "behavio* activation"[Title/Abstract] OR "cognitive restructur*"[Title/Abstract] OR "cognit* training*"[Title/Abstract] OR "behavi* training"[Title/Abstract] OR "iCBT"[Title/Abstract] OR "I-CBT"[Title/Abstract] OR "eCBT"[Title/Abstract] OR "e-CBT"[Title/Abstract] OR "cCBT"[Title/Abstract] OR "c-CBT"[Title/Abstract] OR "Acceptance and Commitment Therapy"[Title/Abstract] OR "Acceptance Commitment Therapy"[Title/Abstract] OR "ACT"[Title/Abstract] OR "Cognitive Behavioral Therapy"[MeSH Terms]	419,848
#4	("randomised"[Title/Abstract] OR "randomized"[Title/Abstract] OR "randomisation"[Title/Abstract] OR "randomization"[Title/Abstract] OR "controlled clinical trial*"[Title/Abstract] OR "placebo*"[Title/Abstract] OR ("random*"[Title/Abstract] AND ("allocat*"[Title/Abstract] OR "assign*"[Title/Abstract])) OR ("blind*"[Title/Abstract] AND ("single"[Title/Abstract] OR "double"[Title/Abstract] OR "treble"[Title/Abstract] OR "triple"[Title/Abstract])))	1,146,116
#5	#1 AND #2 AND #3 AND #4	81

Source: PsycINFO

	Multi-field search	Results
#1	(anxiety or "panic attack" or anxious or depress* or "mood disorder*" or "major depressive disorder" or dysthymia or "affective disorder*").ab. or (anxiety or "panic attack" or anxious or depress* or "mood disorder*" or "major depressive disorder" or dysthymia or "affective disorder*").ti. or ("Depression" or "Anxiety").mh.	540,566
#2	(NLP or "Natural Language Processing" or "large language model" or LLM or GPT or chatbot or "chat-bot" or "machine learning" or "artificial intelligence" or AI or "multimodal large language models" or MLLM or "conversational agent*" or "conversational bot*" or "conversational system*" or "chatterbot*" or "chatter bot*" or "smartbot*" or "smart bot*" or "smart-bot*" or "virtual coach*" or "virtual agent*").ab. or (NLP or "Natural Language Processing" or "large language model" or LLM or GPT or chatbot or "chat-bot" or "conversational agent" or "machine learning" or "artificial intelligence" or AI or "multimodal large language models" or MLLM or "conversational agent*" or "conversational bot*" or "conversational system*" or "chatterbot*" or "chatter bot*" or "smartbot*" or "smart bot*" or "smart-bot*" or "virtual coach*" or "virtual agent*").ti.	28,187
#3	("Cognit* Behavio* Therap*" or "Cognit* Therap*" or "Behavio* Therap*" or CBT or "Dialect* Behavio* Therap*" or DBT or "Metacogniti* Therap*" or "Mind Training" or "Behavio* Activation" or "Cognitive Restructur*" or Cognit* Training* or "Behavi* Training" or "iCBT" or "I-CBT" or "eCBT" or "e-CBT" or "cCBT" or "c-CBT" or "Acceptance and Commitment Therapy" or "Acceptance Commitment Therapy" or ACT).ab. or ("Cognit* Behavio* Therap*" or "Cognit* Therap*" or "Behavio* Therap*" or CBT or "Dialect*	146,765

	Behavio* Therap*" or DBT or "Metacogniti* Therap*" or "Mind Training" or "Behavio* Activation" or "Cognitive Restructur*" or Cognit* Training* or "Behavi* Training" or "iCBT" or "I-CBT" or "eCBT" or "e-CBT" or "cCBT" or "c-CBT" or "Acceptance and Commitment Therapy" or "Acceptance Commitment Therapy" or ACT).ti. or Cognitive Behavioral Therapy.mh.	
#4	randomised OR randomized OR randomisation OR randomization OR "controlled clinical trial*" OR placebo* OR (random* AND (allocat* OR assign*)) OR (blind* AND (single OR double OR treble OR triple))	191,364
#5	#1 AND #2 AND #3 AND #4	40
#6	limit #4 to yr="2016 -Current"	38

Second search

Search conducted on 13th April 2025.

Source: Web of Science Core Collection

	Search strategy (Limit: 2024-11-06 to 3000-12-31)	Results
#1	TS=(anxiety OR "panic attack" OR anxious OR depress* OR "mood disorder*" OR "major depressive disorder" OR dysthymia OR "affective disorder*")	28,540
#2	TS=(NLP OR "Natural Language Processing" OR "large language model" OR LLM OR GPT OR chatbot OR "chat-bot" OR "machine learning" OR "artificial intelligence" OR AI OR "multimodal large language models" OR MLLM OR "conversational agent*" OR "conversational bot*" OR "conversational system*" OR "chatterbot*" OR "chatter bot*" OR "smartbot*" OR "smart bot*" OR "smart-bot*" OR "virtual coach*" OR "virtual agent*")	68,963
#3	TS=("Cognit* Behavio* Therap*" OR "Cognit* Therap*" OR "Behavio* Therap*" OR CBT OR "Dialect* Behavio* Therap*" OR DBT OR "Metacogniti* Therap*" OR "Mind Training" OR "Behavio* Activation" OR "Cognitive Restructur*" OR Cognit* Training* OR "Behavi* Training" OR "iCBT" or "I-CBT" OR "eCBT" OR "e-CBT" OR "cCBT" OR "c-CBT" OR "Acceptance and Commitment Therapy" OR "Acceptance Commitment Therapy" OR ACT)	30,097
#4	TS=(randomised OR randomized OR randomisation OR randomization OR "controlled clinical trial*" OR placebo* OR (random* AND (allocat* OR assign*)) OR (blind* AND (single OR double OR treble OR triple)))	38,965
#5	#1 AND #2 AND #3 AND #4	26

Source: PubMed

	Search strategy	Results
#1	("anxiety"[Title/Abstract] OR "panic attack"[Title/Abstract] OR "anxious"[Title/Abstract] OR "depress*"[Title/Abstract] OR "mood disorder*"[Title/Abstract] OR "major depressive disorder"[Title/Abstract] OR "dysthymia"[Title/Abstract] OR "affective disorder*"[Title/Abstract] OR "Depression"[Mesh] OR "Anxiety"[Mesh])	838,755
#2	("NLP"[Title/Abstract] OR "Natural Language Processing"[Title/Abstract] OR "large language model"[Title/Abstract] OR "LLM"[Title/Abstract] OR "GPT"[Title/Abstract] OR "chatbot"[Title/Abstract] OR "chat-	219,654

	bot"[Title/Abstract] OR "machine learning"[Title/Abstract] OR "artificial intelligence"[Title/Abstract] OR "AI"[Title/Abstract] OR "multimodal large language models"[Title/Abstract] OR "MLLM"[Title/Abstract] OR "conversational agent*"[Title/Abstract] OR "conversational bot*"[Title/Abstract] OR "conversational system*"[Title/Abstract] OR "chatterbot*"[Title/Abstract] OR "smartbot*"[Title/Abstract] OR "smart bot*"[Title/Abstract] OR "smart bot*"[Title/Abstract] OR "virtual coach*"[Title/Abstract] OR "virtual agent*"[Title/Abstract])	
#3	"cognit* behavio* therap*"[Title/Abstract] OR "cognit* therap*"[Title/Abstract] OR "behavio* therap*"[Title/Abstract] OR "CBT"[Title/Abstract] OR "dialect* behavio* therap*"[Title/Abstract] OR "DBT"[Title/Abstract] OR "metacogniti* therap*"[Title/Abstract] OR "Mind Training"[Title/Abstract] OR "behavio* activation"[Title/Abstract] OR "cognitive restructur*"[Title/Abstract] OR "cognit* training*"[Title/Abstract] OR "behavi* training"[Title/Abstract] OR "iCBT"[Title/Abstract] OR "I-CBT"[Title/Abstract] OR "eCBT"[Title/Abstract] OR "e-CBT"[Title/Abstract] OR "cCBT"[Title/Abstract] OR "c-CBT"[Title/Abstract] OR "Acceptance and Commitment Therapy"[Title/Abstract] OR "Acceptance Commitment Therapy"[Title/Abstract] OR "ACT"[Title/Abstract] OR "Cognitive Behavioral Therapy"[MeSH Terms]	429,187
#4	("randomised"[Title/Abstract] OR "randomized"[Title/Abstract] OR "randomisation"[Title/Abstract] OR "randomization"[Title/Abstract] OR "controlled clinical trial*"[Title/Abstract] OR "placebo*"[Title/Abstract] OR ("random*"[Title/Abstract] AND ("allocat*"[Title/Abstract] OR "assign*"[Title/Abstract])) OR ("blind*"[Title/Abstract] AND ("single"[Title/Abstract] OR "double"[Title/Abstract] OR "treble"[Title/Abstract] OR "triple"[Title/Abstract])))	1,180,788
#5	(2024/11/06:3000/12/31[Date – Create])	710,456
#6	#1 AND #2 AND #3 AND #4	15

Source: PsycINFO

	Multi-field search	Results
#1	(anxiety or "panic attack" or anxious or depress* or "mood disorder*" or "major depressive disorder" or dysthymia or "affective disorder*").ab. or (anxiety or "panic attack" or anxious or depress* or "mood disorder*" or "major depressive disorder" or dysthymia or "affective disorder*").ti. or ("Depression" or "Anxiety").mh.	550,864
#2	(NLP or "Natural Language Processing" or "large language model" or LLM or GPT or chatbot or "chat-bot" or "machine learning" or "artificial intelligence" or AI or "multimodal large language models" or MLLM or "conversational agent*" or "conversational bot*" or "conversational system*" or "chatterbot*" or "chatter bot*" or "smartbot*" or "smart bot*" or "smart-bot*" or "virtual coach*" or "virtual agent*").ab. or (NLP or "Natural Language Processing" or "large language model" or LLM or GPT or chatbot or "chat-bot" or "machine learning" or "artificial intelligence" or AI or "multimodal large language models" or MLLM or "conversational agent*" or "conversational bot*" or "conversational system*" or "chatterbot*" or	30,576

	"chatter bot*" or "smartbot*" or "smart bot*" or "smart-bot*" or "virtual coach*" or "virtual agent*").ti.	
#3	("Cognit* Behavio* Therap*" or "Cognit* Therap*" or "Behavio* Therap*" or CBT or "Dialect* Behavio* Therap*" or DBT or "Metacogniti* Therap*" or "Mind Training" or "Behavio* Activation" or "Cognitive Restructur*" or Cognit* Training* or "Behavi* Training" or "iCBT" or "I-CBT" or "eCBT" or "e-CBT" or "cCBT" or "c-CBT" or "Acceptance and Commitment Therapy" or "Acceptance Commitment Therapy" or ACT).ab. or ("Cognit* Behavio* Therap*" or "Cognit* Therap*" or "Behavio* Therap*" or CBT or "Dialect* Behavio* Therap*" or DBT or "Metacogniti* Therap*" or "Mind Training" or "Behavio* Activation" or "Cognitive Restructur*" or Cognit* Training* or "Behavi* Training" or "iCBT" or "I-CBT" or "eCBT" or "e-CBT" or "cCBT" or "c-CBT" or "Acceptance and Commitment Therapy" or "Acceptance Commitment Therapy" or ACT).ti. or Cognitive Behavioral Therapy.mh.	149,303
#4	randomised OR randomized OR randomisation OR randomization OR "controlled clinical trial*" OR placebo* OR (random* AND (allocat* OR assign*)) OR (blind* AND (single OR double OR treble OR triple))	210,704
#5	#1 AND #2 AND #3 AND #4	43
#6	limit #4 to yr="2024 -Current"	10
Removed duplicates with the first search due to “by year” limit on PsycINFO OVID		3

Supplementary Table 3: Eligibility criteria for study inclusion

	Criteria	Inclusion Criteria	Exclusion Criteria
P	All populations	All demographic groups were considered eligible.	None.
I	Use of an AI-delivered CBT intervention	Only studies utilizing AI-delivered CBT interventions, or variations of CBT, were included. In this study, AI-delivered CBT interventions were defined as software-based agents or bots using AI models to administer and/or conduct the therapy, in order to achieve therapeutic outcomes for participants.	Interventions that utilized AI for purposes other than delivering the CBT intervention itself were excluded, such as AI chatbots that motivated participants to adhere to the intervention, or offered only emotional support. Interventions that utilized machine learning to assign participants to different CBT interventions were also excluded.
C	All forms of comparison were included	Studies with any form of comparison were included, whether that was standard care or an alternative CBT intervention.	None.
O	Anxiety and/or depressive symptoms	Any outcomes related to anxiety or depressive symptoms qualified, whether measured by self-reported surveys, objective indicators (e.g., passive sensing systems like audio or visual data), or third-party assessments.	Outcomes that did not measure anxiety or depressive symptoms in any form were excluded.
T	Only studies published on or after 2016-01-01 till now were included	This search only included articles that were published on or after 2016-01-01 up till “current”. In some search engines, 3000-12-31 was used as the “end date”.	Any articles published before 2016-01-01 were excluded.
S	Randomized Controlled Trials (RCTs)<br><br>Peer-reviewed articles<br><br>Articles written in English	Only Randomized Controlled Trials (RCTs) were included.<br><br>Only peer-reviewed articles were included.<br><br>Only articles published in English were included. Articles that had an English translation were also included.	This review excluded quasi-experimental studies that lacked a control arm or randomization groups. Additionally, it excluded narrative reviews, systematic reviews, scoping reviews, and other forms of non-original research designs. This review also excluded pilot studies.<br><br>Proceedings, pre-prints, conference posters, and similar materials were not included.<br><br>Articles with no form of published English translation were not included.

Supplementary Data 1: Extraction table and Quality assessment

Attached please find an excel spreadsheet.
